# Combined effects of smoking and HIV infection on the occurrence of aging-related manifestations

**DOI:** 10.1038/s41598-023-39861-5

**Published:** 2023-12-08

**Authors:** Laurent Boyer, Sonia Zebachi, Sébastien Gallien, Laurent Margarit, Bruno Ribeiro Baptista, José-Luis Lopez-Zaragoza, Thomas D’Humières, Françoise Zerah, Sophie Hue, Geneviève Derumeaux, Serge Adnot, Etienne Audureau, Jean-Daniel Lelièvre

**Affiliations:** 1 Département de Physiologie-Explorations Fonctionnelles, FHU Senec, APHP Hôpital Henri Mondor, 1 rue Gustave Eiffel, 94010 Créteil, France; 2 Univ Paris Est Creteil, INSERM IMRB, UMR U955, 94010 Créteil, France; 3 Service de maladies infectieuses et immunologie clinique, APHP Hôpital Henri Mondor, 94010 Créteil, France; 4 Faculté de médecine, Université Paris Est (UPEC), 94010 Créteil, France; 5 Service d’Immunologie Biologique, APHP Hôpital Henri Mondor, 94010 Créteil, France; 6 Service de Santé Publique, APHP Hôpital Henri Mondor, 94010 Créteil, France

**Keywords:** HIV infections, Medical research, Epidemiology, Risk factors

## Abstract

Both HIV-1 infection and smoking may contribute to the development of ageing-related manifestations affecting the prognosis of people living with HIV, but it is unclear whether HIV and smoking exert their effects independently or interact by potentiating each other. We conducted a cross-sectional study in 192 people living with HIV aged- and gender-matched with 192 HIV-uninfected controls, assessing the relative effect of HIV-1/smoking status on lung function (FEV1), bone mineral density (BMD), appendicular skeletal muscle mass index (ASMI), aortic pulse-wave velocity (PWV), insulin resistance (HOMA-IR) and renal function. In both unadjusted and adjusted analyses, FEV1, BMD and ASMI significantly differed according to smoking/HIV status, with the worst parameters found in HIV-1 infected patients currently smoking, and BMD and ASMI decreased to a lesser extent in HIV-1 infected patients formerly smoking (> 10 pack-years). Values in people living with HIV with < 10 pack-years exposure were of similar magnitude to those from controls. Regarding PWV, HOMA-R and eGFR, no significant differences were found, with the exception of eGFR values which were globally lower in HIV-1 infected patients. In conclusion HIV infection and smoking acted synergistically and were associated with a wasting phenotype combining muscle mass and bone mineral reduction.

Clinical Trial Registration (registrar, website, and registration number), where applicable: CPP 10-023, 09-027, 10-034.

## Introduction

With the advent of combined antiretroviral therapy (ART), people living with HIV (PLWH) live longer, currently reaching a median age higher than 50 years^1^. However, PLWH still die earlier than non-infected patients, mainly due the development of aging related comorbidities that adversely affect the prognosis of the disease, such as chronic obstructive pulmonary diseases, cardiovascular diseases, diabetes, renal insufficiency, or osteoporosis. These comorbidities are each individually associated with worse quality of life or increased mortality^2–7^. Decreased limb muscle and increased central adiposity are associated with 5-year all-cause mortality in HIV infection^8^. However, whether such systemic effects are ascribable directly to HIV disease and ART, or to other factors such as aging, environmental or behavioral determinants is still in debate. Among these factors, several are modifiable risk factors for comorbidities and it is crucial to determine whether actions reducing these risk factors may be sufficient to prevent or reverse the development of these comorbidities.

Tobacco smoking is the main modifiable risk that has a strong impact on age related comorbidities in the general population, in particular regarding lung and cardiovascular disease or osteoporosis. The systemic effects of smoking are mainly represented by pulmonary alterations such as emphysema and chronic obstructive pulmonary disease^9–12^. The higher prevalence of smoking among PLWH compared to the general population has led to an increasing cumulative exposure to tobacco in this population^13,14^. However, whether smoking is the main driver of age related diseases and comorbidities in PLWH is still a subject of debate^14,15^. If the relationship between smoking and cardiovascular diseases such as atherosclerosis and myocardial infarction, may be stronger in PLWH than in uninfected subjects^16^, we do not know how HIV affects the relationship between smoking and the other systemic manifestations associated with cigarette smoke exposure. Similarly, we do not know whether HIV and smoking may exert their effects independently or may interact by potentiating each other.

To further our understanding of the impact of tobacco smoking to the age-related systemic manifestations in HIV-infected individuals, we investigated the association between smoking and several parameters such as arterial stiffness, bone mineral density, muscle mass, insulin resistance and kidney function, in PLWH and uninfected individuals and determined whether these relationships differed depending on HIV status. Because smoking may gradually exert its potential systemic effects within a continuum, relevant associations may be overlooked when only focusing on clinically established diseases. We consequently investigated these complex associations using continuous biological and functional parameters operating also at earlier stages of disease development.

## Methods

### Study design and participants

Participants living with HIV were recruited from the CARDAMONE study, a cross-sectional monocentric study of adult PLWH enrolled from the HIV outpatient clinic of the Henri Mondor Teaching hospital, France, between 2009 and 2012. To be included, patients had to have plasma HIV RNA below 50 copies/ml under c-ART and no past major cardiovascular event (i.e. myocardial infarction/chronic heart failure). HIV-uninfected individuals were recruited from the Clinical Investigation Center of the Henri Mondor Teaching hospital, as previously described^9,10,17^. For the present analysis, HIV-infected patients were 1:1 gender- and age-matched (using 5-years classes) to HIV-uninfected patients. A comparison of the main characteristics of subjects matched with those unmatched and discarded from the present analysis is shown in Supplemental Tables 1 and 2, indicating notable age-related differences between (un)matched subjects, with the youngest PLWH and the oldest controls being left out of the analysis. All studies were approved by the ethical committee of the Henri-Mondor Teaching Hospital (CARDAMONE: CPP 10-023; uninfected individuals: CPP 09-027 and 10-034). All participants provided written informed consent before inclusion. All research was performed in accordance with the Declaration of Helsinki.

### Variables and data measurement

Demographic, clinical and lifestyle factors were collected for all participants from medical records, including age, gender, smoking, body mass index, waist circumference and blood pressure. Smokers were defined as individuals who had smoked more than 100 cigarettes in their lifetime^18^, distinguishing between current and former (≥ 1 year) smokers who had quit smoking at the time of the study.

Each participant underwent spirometry, plethysmography measurement according to ATS/ERS consensus guidelines^19^. In each participant, arterial stiffness (aortic pulse-wave velocity, PWV) was measured as the carotid-femoral pulse-wave velocity using the Complior Analyse device (Alam Medical, Vincennes, France). Bone mineral density (BMD) at the hip (femoral neck) and lumbar spine was determined using dual-energy X-ray absorptiometry (Lunar iDXA, GE Healthcare, UK). BMD is reported as the absolute value (g/cm^2^). T-scores were computed to classify participants as having normal BMD or osteoporosis (defined as T-score < − 2.5 at either site). To assess muscle mass, appendicular skeletal muscle mass (ASM) was measured as the fat-free soft-tissue masses of the arms and legs divided by height squared and ASM index (ASMI) was then computed as ASMM divided by height squared. The cutoff for defining sarcopenia was two standard deviations below the mean sex-specific ASMI values in the Rosetta Study of young adults (5.45 for females and 7.26 for males), as proposed by Baumgartner et al.^20^. Insulin resistance was assessed by calculating the homeostasis model assessment of insulin resistance (HOMA-IR) (insulin·glucose)/22.5), and renal function by estimating the glomerular filtration rate (eGFR) using the Cockcroft-Gault formula. Other biological data included hemoglobin, white blood cell count (WBC), fasting glycemia, Hba1c, cholesterol (total, HDL, LDL), triglycerides, CRP and specifically in PLWH T lymphocytes parameters (i.e. Nadir CD4^+^ cell count, CD4^+^ and CD8^+^ cell counts, CD4^+^/CD8^+^ ratio).

### Statistical analysis

Qualitative variables are reported as numbers and percentages, and quantitative variables as means (± standard deviation, SD) or medians [interquartile range, IQR], depending on the normality of variable distributions as assessed by Shapiro–Wilk tests. Unadjusted between-groups comparisons were performed by means of mixed effects regression models to account for the 1:1 matching between PLWH and HIV-uninfected patients, using linear regression for continuous parameters and logistic regression for binary variables. Mixed effects linear multivariate models adjusted for age and gender were secondarily conducted to assess the relative effects of smoking and HIV-infection on aging-related systemic manifestations (i.e. arterial stiffness, bone mineral density, muscle mass, insulin resistance and kidney function). To assess the potential effect of the combination between smoking status and HIV, a composite 6-categories variable was entered in to the model, as follows: controls who were (i) never smokers or < 10 pack-years, (ii) former smokers with > 10 pack-years or (iii) current smokers with > 10 pack-years; and HIV-1-infected patients who were (iv) never smokers or < 10 pack-years, (v) former smokers with > 10 pack-years or (vi) current smokers with > 10 pack-years. No adjustment for multiple testing was done in the present study. Analyses of the effects of smoking and HIV-1 status were exploratory by nature and performed on prespecified ageing parameters of interest.

For illustrative purposes, a Gabriel’s biplot was created to project the subjects along the principal components axes from a principal components analysis (PCA) based on their individual aging-related characteristics^21^. HIV/smoking 6-categories status was then mapped on the biplot by attributing different colors to patient’s groups. Missing data for the main outcomes and covariates ranged from 0 to 13% (ASMI); all analyses were performed on complete cases using Stata v16.0 (StataCorp, College Station, TX, USA) and data visualizations using R v3.6.2 (R Foundation, Vienna, Austria).

### Ethics approval and consent to participate

All studies were approved by the ethical committee of the Henri-Mondor Teaching Hospital (CARDAMONE: CPP 10-023; uninfected individuals: CPP 09-027 and 10-034). All participants provided written informed consent before inclusion.

## Results

### HIV-infected and matched controls differed on key baseline characteristics

From an initial total of 629 patients (N = 239 PLWH and N = 390 HIV-uninfected controls), 1:1 age- and gender-matching was possible for 378 patients (189 patients in each subgroup). Main characteristics of the participants are described in Table 1. In addition to age (overall mean 49.8 ± 8.2 years) and gender (overall 21.2% women), matched participants were also comparable regarding systolic blood pressure, pulse-wave velocity, HOMA-IR, and the ratio forced expiratory volume in one second (FEV1)/forced vital capacity (FVC). Overall, PLWH were characterized by a higher proportion of current smokers and sarcopenia, lower body mass index (BMI), eGFR and musculoskeletal parameters (i.e. hip and lumbar BMD, ASMI) compared to non HIV-infected subjects. No statistically significant difference was found between groups regarding mean past cigarette smoke exposure as expressed in pack-years.

**Table 1:** Baseline characteristics of the study population.

	N completed	Controls N = 189	People living with HIV N = 189	p-value*
Age, years	378	50.0 ± 8.4	49.6 ± 8.0	0.644
Gender, women (%)	378	40 (21.2%)	40 (21.2%)	1.000
Smoking status	378			0.043
Never smoker (%)	180	100 (52.9%)	80 (42.3%)	
Former smoker (%)	78	40 (21.2%)	38 (20.1%)	
Current smoker (%)	120	49 (25.9%)	71 (37.6%)	
Pack-years of cigarettes	378	12.7 (± 18.3)	12.3 (± 14.9)	0.793
Smoking/Pack-years status	378			0.445
Never smokers or < 10 Pack-years	214	111 (58.7%)	103 (54.5%)	
> 10 Pack-years, former smokers	61	32 (16.9%)	29 (15.3%)	
> 10 Pack-years, current smokers	103	46 (24.3%)	57 (30.2%)	
BMI, kg/m^2^	376	26.9 (± 3.6)	24.1 (± 3.9)	< 0.001
Obesity	376	35 (18.6%)	18 (9.6%)	0.013
Dyslipidemia	355	53 (30.5%)	76 (42.0%)	0.024
Diabetes	369	3 (1.6%)	8 (4.4%)	0.126
Systolic blood pressure, mmHg	346	120.3 (± 14.4)	121.7 (± 14.2)	0.392
Diastolic blood pressure, mmHg	346	78.4 (± 8.6)	76.7 (± 9.7)	0.080
HTA	345	19 (11.7%)	27 (14.8%)	0.387
FEV1, % predicted	347	101.5 (± 15.3)	98.4 (± 17.3)	0.078
FEV1/FVC	347	81.8 (± 6.5)	81.5 (± 7.8)	0.750
Pulse-wave velocity, m/s	341	10.5 (9.4; 11.6)	10.2 (9.5; 11.6)	0.892
BMD total lumbar, g/cm^2^	348	1.2 (± 0.2)	1.1 (± 0.2)	0.002
BMD hip (lowest), g/cm^2^	347	1.0 (± 0.2)	1.0 (± 0.2)	0.002
ASMI, kg/m^2^	330	8.2 (± 1.3)	7.7 (± 1.3)	0.001
Sarcopenia (%)	330	4 (2.7%)	41 (22.8%)	< 0.001
HOMA-IR	353	2.0 (1.3; 3.5)	2.3 (1.5; 3.3)	0.593
Glomerular flow rate, mL/min	362	98.2 (86.5; 116.1)	92.5 (81.4; 110.9)	0.026
Time since HIV diagnosis, years	189	–	12.6 (8.7; 18.4)	–
History of AIDS (%)	189	–	51 (27.0%)	–
Nadir CD4^+^ cell count, cells/mm^3^	185	–	142.0 (35.0; 244.0)	–
CD4^+^ cell count, cells/mm^3^	174	–	237.5 (79.0; 404.0)	–
CD8^+^ cell count, cells/mm^3^	188	–	645.0 (478.0; 842.0)	–
CD4^+^/CD8^+^ ratio	188	–	0.8 (0.6; 1.1)	–
ART use at enrollment	182			
PI-based therapy		88 (48.4%)		–
INI-based triple therapy		10 (5.5%)		–
RTI-based triple therapy		77 (42.3%)		–
Others		3 (1.6%)		
No treatment		4 (2.2%)		–

*p-values from mixed effects linear or logistic regression model accounting for matching between HIV-infected and HIV-uninfected patients.

Results are mean (± standard deviation), median (interquartile range) or N (%).

All PLWH had plasma HIV RNA below 50 copies/ml, of whom 98% were receiving ART. The median nadir CD4^+^ T-cell count was 142 cells/mm^3^ (IQR, 35; 244 cells/mm^3^), the current CD4^+^ T-cell count was 237.5 (IQR, 79; 404), the baseline median CD4^+^/CD8^+^ ratio was 0.82 (IQR 0.58; 1.14) and 27% had a history of AIDS.

### Effects of combined smoking and HIV status on ageing-related parameters

Results from unadjusted and age–gender adjusted linear regression modeling are shown in Table 2 (FEV1, BMD, ASMI) and Table 3 (PWV, HOMA-R, eGFR).

**Table 2:** Effects of smoking and HIV-1 status on aging-related parameters: FEV1, BMD and ASMMI.

Ageing-related parameter	Group			Unadjusted analysis			Adjusted analysis*		
				Beta coefficient (CI 95%)	p-value	p-value (overall)	Beta coefficient (CI 95%)	p-value	p-value (overall)
FEV_1_	Controls	< 10 PY		0 (ref)	–	**0.044**	0 (ref)	–	0.054
		> 10 PY	Former smokers	1.53 (− 5.36; 8.43)	0.663		1.31 (− 5.62; 8.23)	0.712	
			Current smokers	− 1.83 (− 7.64; 3.98)	0.537		− 1.83 (− 7.75; 4.10)	0.545	
	HIV	< 10 PY		− 1.47 (− 5.92; 2.98)	0.518		− 1.39 (− 5.86; 3.08)	0.542	
		> 10 PY	Former smokers	− 0.26 (− 7.00; 6.49)	0.940		− 0.56 (− 7.37; 6.24)	0.871	
			Current smokers	− 8.11 (− 13.36; − 2.85)	**0.003**		− 8.03 (− 13.29; − 2.77)	**0.003**	
BMD Hip	Controls	< 10 PY		0 (ref)	–	**0.0002**	0 (ref)	–	**< 0.0001**
		> 10 PY	Former smokers	0.00 (− 0.07; 0.07)	0.999		0.01 (− 0.06; 0.07)	0.868	
			Current smokers	− 0.05 (− 0.10; 0.01)	0.110		− 0.02 (− 0.07; 0.04)	0.524	
	HIV	< 10 PY		− 0.03 (− 0.07; 0.01)	0.180		− 0.03 (− 0.07; 0.02)	0.230	
		> 10 PY	Former smokers	− 0.09 (− 0.15; − 0.02)	**0.007**		− 0.08 (− 0.14; − 0.01)	**0.015**	
			Current smokers	− 0.11 (− 0.16; − 0.06)	**< 0.0001**		− 0.12 (− 0.17; − 0.07)	**< 0.0001**	
ASMI	Controls	< 10 PY		0 (ref)	–	**< 0.0001**	0 (ref)	–	**< 0.0001**
		> 10 PY	Former smokers	0.26 (− 0.31; 0.83)	0.363		0.27 (− 0.23; 0.77)	0.285	
			Current smokers	− 0.44 (− 0.89; 0.00)	0.051		− 0.09 (− 0.48; 0.31)	0.659	
	HIV	< 10 PY		− 0.31 (− 0.64; 0.01)	0.061		− 0.23 (− 0.53; 0.07)	0.136	
		> 10 PY	Former smokers	− 0.68 (− 1.17; − 0.19)	**0.007**		− 0.72 (− 1.16; − 0.28)	**0.001**	
			Current smokers	− 0.96 (− 1.34; − 0.58)	**< 0.0001**		− 1.05 (− 1.40; − 0.70)	**< 0.0001**	

*Mixed effects linear regression model adjusted for age and gender.

Significant values are in bold.

**Table 3:** Effects of smoking and HIV status on aging-related parameters: PWV, HOMA-R and eGFR.

Ageing-related parameter	Group			Unadjusted analysis			Adjusted analysis*		
				Beta coefficient (CI 95%)	p-value	p-value (overall)	Beta coefficient (CI 95%)	p-value	p-value (overall)
PWV	Controls	< 10 PY		0 (ref)	–	0.148	0 (ref)	–	0.684
		> 10 PY	Former smokers	0.64 (− 0.14; 1.43)	0.108		0.42 (− 0.33; 1.16)	0.274	0
			Current smokers	0.38 (− 0.30; 1.07)	0.272		0.19 (− 0.47; 0.85)	0.576	
	HIV	< 10 PY		0.22 (− 0.31; 0.74)	0.414		0.19 (− 0.32; 0.70)	0.472	
		> 10 PY	Former smokers	0.94 (0.14; 1.74)	**0.022**		0.60 (− 0.17; 1.37)	0.126	
			Current smokers	− 0.07 (− 0.70; 0.55)	0.820		0.06 (− 0.54; 0.66)	0.836	
HOMA-R	Controls	< 10 PY		0 (ref)	–	0.101	0 (ref)	–	0.193
		> 10 PY	Former smokers	0.96 (− 0.28; 2.20)	0.129		0.82 (− 0.41; 2.06)	0.192	
			Current smokers	− 0.63 (− 1.76; 0.49)	0.271		− 0.72 (− 1.86; 0.41)	0.212	
	HIV	< 10 PY		− 0.14 (− 0.97; 0.70)	0.748		− 0.15 (− 0.98; 0.69)	0.730	
		> 10 PY	Former smokers	0.77 (− 0.48; 2.02)	0.226		0.55 (− 0.71; 1.81)	0.393	
			Current smokers	− 0.66 (− 1.65; 0.33)	0.191		− 0.62 (− 1.60; 0.36)	0.217	
eGFR (Cockcroft)	Controls	< 10 PY		0 (ref)	–	0.110	0 (ref)	–	**0.027**
		> 10 PY	Former smokers	6.67 (− 3.10; 16.44)	0.181		7.40 (− 1.49; 16.29)	0.103	
			Current smokers	3.07 (− 5.89; 12.03)	0.502		7.56 (− 0.66; 15.78)	0.071	
	HIV	< 10 PY		− 5.24 (− 11.49; 1.01)	0.100		− 3.50 (− 9.46; 2.47)	0.250	
		> 10 PY	Former smokers	− 2.14 (− 11.92; 7.65)	0.668		0.43 (− 8.70; 9.57)	0.926	
			Current smokers	− 2.72 (− 10.35; 4.91)	0.485		− 4.00 (− 11.11; 3.11)	0.270	

*Mixed effects linear regression model adjusted for age and gender.

Significant values are in bold.

In both unadjusted and adjusted analyses, FEV1, BMD and ASMI significantly differed according to smoking/HIV status (Table 2), with the worst parameters significantly found in PLWH currently smoking (adjusted regression coefficients compared to controls never smokers or < 10 pack-years: FEV1 − 8.03, p = 0.003; BMD − 0.12, p < 0.0001; ASMI − 1.05, p < 0.0001). BMD and ASMI were also significantly decreased in HIV-1 infected patients formerly smoking, but to a lesser extent (BMD − 0.08, p = 0.014; ASMI − 0.72, p = 0.001). Of note, values for these parameters did not substantially differ in controls according to smoking status. Likewise, values in PLWH who were never smokers or with < 10 pack-years were of similar magnitude to those from controls.

Regarding PWV, HOMA-R and eGFR (Table 3), no significant differences were found between smoking/HIV categories in all unadjusted and adjusted analyses, to the exception of eGFR values which were substantially lower in PLWH to those from controls.

**Figure 1:**
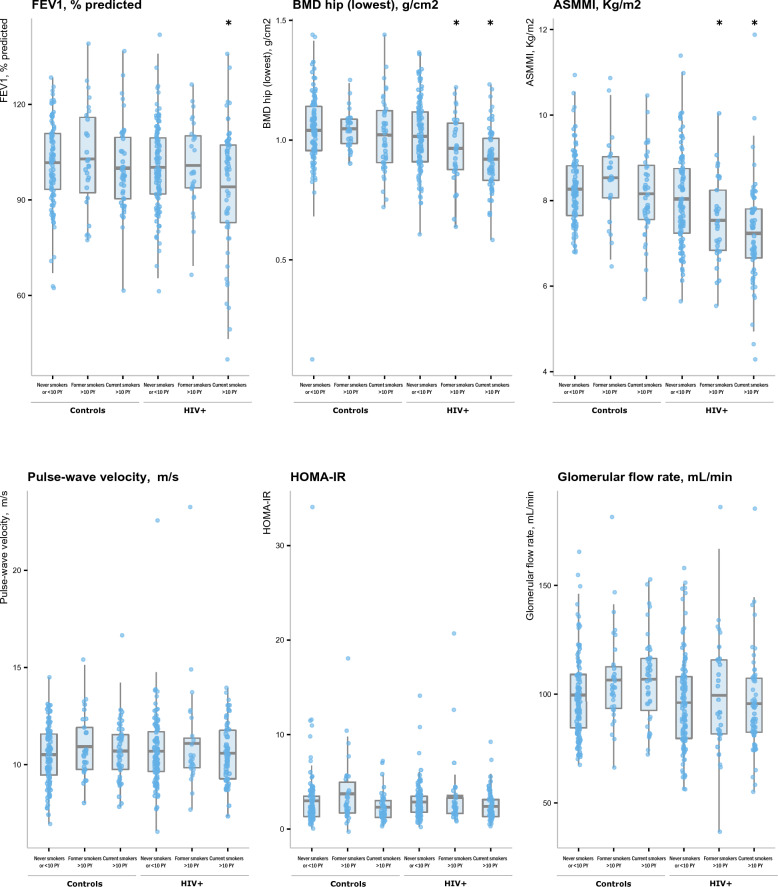
Boxplots of ageing-related parameters according to smoking and HIV+ status. Results are shown as boxplots, with each box representing the interquartile range (1st to 3rd quartile, IQR), the line within the box indicating the mean, and the whiskers extending to 1.5 times the IQR above and below the box; the dots represent individual values for each subject as predicted from mixed effects linear regression modeling adjusted for age and gender. Asterisks (*) indicate subgroups statistically significantly different from never smoker controls.

To further illustrate these findings, Fig. 1 shows as boxplots the age–gender adjusted comparisons of the ageing-related parameters values according to the composite smoking-HIV status, confirming the decreased FEV, BMD and ASMI values found in PLWH currently smoking and, to a lesser extent, formerly smoking for BMD and ASMMI. Detailed statistics including raw and adjusted means are given in Supplemental Table 3.

**Figure 2:**
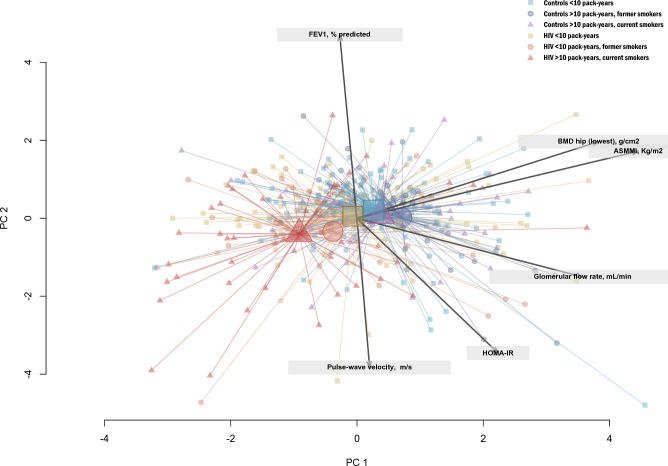
2-dimensional biplot representation of patients’ characteristics according to smoking and HIV+ status. Biplot representation allows the visualization of relationships between ageing parameters (arrows) while simultaneously displaying the patients (dots), based on their individual characteristics. Results are projected onto the two first dimensions generated by principal component analysis. Colors for observations correspond to one of the six groups according to HIV and smoking status (i.e. controls who were (i) never smokers or < 10 pack-years, (ii) former smokers with > 10 pack-years or (iii) current smokers with > 10 pack-years; and people living with HIV who were (iv) never smokers or < 10 pack-years, (v) former smokers with > 10 pack-years or (vi) current smokers with > 10 pack-years). Highlighted markers of increased size within each group represent the group centroid of the group.

Figure 2 shows the 2-dimensional biplot representation of patients’ characteristics according to the composite smoking and HIV+ status variable. PLWH currently or, to a lesser extent, formerly smoking were distinctively projected in the left area of the plot, indicating lower values in ASMI and BMD, while controls and PLWH who were never smokers or with < 10 pack-years were all closely located in the middle-right area, indicating a global overlap in characteristics.

## Conclusion

The main finding of this study is that HIV infection and smoking interact by potentiating each other’s negative effects on ageing. This deleterious effect concerns lung function, bone mineral density and muscle mass, with worse parameters found in PLWH currently smoking. Our findings strongly suggest that smoking acts synergistically with HIV infection to develop aging-related complications.

The synergic effect of cigarette smoke and HIV infection is particularly observed on bone mineral density and muscle mass, that is also linked with low BMI. As others, we observed that bone density and muscle mass were lower in PLWH^22–25^. In large cohort studies, HIV infection was shown to be independently associated with low bone mineral density, and this association remained despite adjustment for traditional risk factors, in particular smoking status^23^. However, whether smoking and HIV-1 infection effects are cumulative and/or whether smoking effects may differ between PLWH and HIV non-infected individuals was not determined in these different studies. We observed that low bone density and low muscle mass are features of the same group of patients, suggesting a common phenomenon leading to a progressive wasting of muscle tissue and bone minerals, and a wasting profile^26,27^. This observation may be due to the lower BMI observed in PLWH compared to the others and may depend on the choice of the control population that has higher BMI. Moreover, as in smokers with or without chronic obstructive pulmonary disease (COPD), low bone mineral density and muscle mass are associated with a lower diffusion capacity and probably with emphysema^11^.

Our results are a new piece of evidence of the synergistic effect of HIV-1 and cigarette smoke on lung function as suspected by the multiple biological changes described along the pulmonary tree when these two factors are combined^28^. This may partially explain the higher decline of lung function described in HIV current smokers than HIV non-smokers^29^, in a population of patient with an already known higher prevalence of airways obstruction than non-HIV infected subjects^30,31^.

Regarding arterial stiffness, no differences were found between smoking and HIV categories in all unadjusted and adjusted analyses. Arterial stiffness assessed by PWV is a sub-clinical marker of atherosclerosis that is associated with increased of cardiovascular events and death both in the general population and in PLWH^3^. Whether people chronically living with HIV have a higher level of pulse wave velocity than non-HIV subjects is object of debate and may depend on the population^32^. However, patients receiving ART and with a suppressed viral replication at the time of pulse wave velocity measurement as in our study, did not present a higher arterial stiffness than non-infected individuals^32^. Our data contrasts with previous studies showing that smoking was more strongly associated with carotid intima-media thickness and myocardial infarction in PLWH compared with HIV-uninfected subjects^16,33^. These differences may be essentially linked to our inclusion criteria: we explored our population at a preclinical stage under the level of cardiovascular disease, since none of the PLWH had presented any cardiovascular events.

One of the strengths of our study is the evaluation of several systemic manifestations concomitantly and objectively quantified. To date, most studies on the impact of comorbidities in PLWH used data on self-reported concurrent chronic conditions or assessed individually. Most systemic manifestations have been studied separately, whereas most HIV infected patients may have two or more chronic morbidities^15^. Interestingly we observed that the expression of manifestations induced by cigarette smoking differed depending on the HIV status, some were amplified and other were not modulated by the chronic infection. More interestingly, smoking combined with HIV was mainly associated with a special cluster of systemic manifestations combining a bone and muscle wasting profile with lung alterations. Similarly, bone, muscle and lung profile in response to cigarette smoke exposure seemed not to be associated with increase arterial stiffness suggesting a different pathophysiological process leading to this alteration in this population, and that different mechanism may be involved in this different manifestation. Our study has also limitations worth mentioning. Sample sizes in HIV/smoking subgroups were somewhat low (ranging from 29 to 111), thus potentially limiting the statistical power of the study to identify statistically significant relationships. It should also be noticed that PLWH included in our study were restricted to those patients with undetected viral load and without overt cardiac comorbidity, and that some individuals from the youngest and oldest age groups were discarded from the analysis due to the age–gender matching procedure, thus potentially limiting the generalizability of our results to broader populations. Finally, adjustment for BMI or other cardiovascular risk factors was not performed considering their potential high level of correlations with ageing parameters (e.g. BMI and ASMMI/sarcopenia; HOMA-IR and diabetes. Given their potential intermediate role in the causal chain between smoking/HIV and ageing parameters, a mediation analysis would have been of interest but was not performed due to the limited sample size of our study to test such more complex relationships.

In conclusion, we find a combined effect of smoking and HIV infection on age related systemic manifestations and HIV appeared as an additive risk factor for some cigarette smoke induced systemic manifestations. Smoking and HIV may be mainly associated with a wasting phenotype associated with lung alterations in HIV infected individuals. These data emphasize again the need to integrate actively smoking cessation in health policies for PLWH, but also to personalize the HIV smoker’s health management with nutrition and exercise to prevent or reverse the bone and muscle loss.

More globally, these emphasize the need to target modifiable risk factors to prevent comorbidities in PLWH. Given the high prevalence of tobacco use in people living with HIV in both high-income and low or middle-income countries, policies and practices to promote tobacco cessation have to be a central strategy to improve the health outcomes in this population.

### Supplementary Information

Supplementary Tables.

## Data Availability

The datasets used and/or analysed during the current study are available from the corresponding author on reasonable request.
