# Supplementary material for: Combined effects of smoking and HIV infection on the occurrence of aging-related manifestations
Source: Sci Rep. 2023 Dec 8;13:21745. doi: 10.1038/s41598-023-39861-5 (PMC10709459; doi:10.1038/s41598-023-39861-5)
Supplement: Supplementary file 1 — Supplementary Tables. [file 41598_2023_39861_MOESM1_ESM.pdf]

Supplemental Table 1. Comparison of main characteristics between matched and unmatched patients: HIV-1 infected subjects

	Matched	Unmatched	
	N=189	N=50	
	**Estimate**	**Estimate**	**p-value**
Age, years	49.6 (±8.0)	39.7 (±5.3)	**<0.001**
Gender, women (%)	40 (21.2%)	42 (84.0%)	**<0.001**
Smoking status			
Never smoker (%)	80 (42.3%)	22 (44.0%)	0.603
Former smoker (%)	38 (20.1%)	7 (14.0%)	
Current smoker (%)	71 (37.6%)	21 (42.0%)	
Pack-years of cigarettes	12.3 (±14.9)	9.3 (±13.8)	0.204
Smoking / Pack-years status			
Never smokers or <10 Pack-years	103 (54.5%)	35 (70.0%)	0.093
>10 Pack-years, former smokers	29 (15.3%)	3 (6.0%)	
>10 Pack-years, current smokers	57 (30.2%)	12 (24.0%)	
BMI, kg/m2	24.1 (±3.9)	24.5 (±5.7)	0.602
Obesity	18 (9.6%)	10 (20.0%)	**0.042**
Dyslipidemia	76 (42.0%)	13 (26.5%)	**0.049**
Diabetes	8 (4.4%)	0 (0.0%)	0.208
Systolic blood pressure, mmHg	121.7 (±14.2)	116.6 (±10.5)	**0.020**
Diastolic blood pressure, mmHg	76.7 (±9.7)	74.8 (±8.8)	0.202
HTA	27 (14.8%)	3 (6.1%)	0.150
FEV1, % predicted	98.4 (±17.3)	93.3 (±12.5)	0.054
FEV1/FVC	81.5 (±7.8)	85.4 (±8.4)	**0.003**
Pulse-wave velocity, m/s	10.2 (9.5;11.6)	9.7 (8.8;10.4)	**<0.001**
BMD total lumbar, g/cm2	1.1 (±0.2)	1.2 (±0.2)	**0.048**
ASMI, Kg/m2	7.7 (±1.3)	6.9 (±1.4)	**0.001**
Sarcopenia (%)	41 (22.8%)	0 (0.0%)	**0.001**
HOMA-IR	2.3 (1.5;3.3)	2.0 (±1.3)	**0.033**
Time since HIV diagnosis, years	12.6 (8.7;18.4)	11.4 (8.9;17.6)	0.477
History of AIDS (%)	51 (27.0%)	10 (20.0%)	0.314
Nadir CD4+ cell count, cells/mm3	142.0 (35.0;244.0)	177.0 (102.0;272.5)	0.123
CD4+ cell count, cells/mm3	237.5 (79.0;404.0)	292.5 (148.0;488.0)	0.145
CD8+ cell count, cells/mm3	645.0 (478.0;842.0)	572.5 (393.0;750.0)	0.053
CD4+/CD8+ ratio	0.8 (0.6;1.1)	1.0 (0.8;1.2)	**0.045**

Results are mean (±standard deviation), median (interquartile range) or N(%)

Supplemental Table 2. Comparison of main characteristics between matched and unmatched patients: controls

	Matched	Unmatched	
	N=189	N=201	
	**Estimate**	**Estimate**	**p-value**
Age, years	50.0 (±8.4)	59.8 (±7.1)	**<0.001**
Gender, women (%)	40 (21.2%)	95 (47.3%)	**<0.001**
Smoking status			
Never smoker (%)	101 (53.4%)	94 (47.5%)	0.487
Former smoker (%)	39 (20.6%)	48 (24.2%)	
Current smoker (%)	49 (25.9%)	56 (28.3%)	
Pack-years of cigarettes	12.7 (±18.3)	14.7 (±20.2)	0.318
Smoking / Pack-years status			
Never smokers or <10 Pack-years	111 (58.7%)	109 (54.2%)	0.601
>10 Pack-years, former smokers	32 (16.9%)	41 (20.4%)	
>10 Pack-years, current smokers	46 (24.3%)	51 (25.4%)	
BMI, kg/m2	26.9 (±3.6)	26.6 (±4.0)	0.477
Obesity	35 (18.6%)	33 (16.6%)	0.599
Dyslipidemia	53 (30.5%)	38 (19.9%)	**0.020**
Diabetes	3 (1.6%)	5 (2.5%)	0.725
Systolic blood pressure, mmHg	120.3 (±14.4)	125.4 (±14.6)	**0.001**
Diastolic blood pressure, mmHg	78.4 (±8.6)	76.2 (±8.2)	**0.013**
HTA	19 (11.7%)	29 (15.3%)	0.315
FEV1, % predicted	101.5 (±15.3)	101.5 (±18.6)	0.996
FEV1/FVC	81.8 (±6.5)	80.0 (±7.2)	**0.017**
Pulse-wave velocity, m/s	10.5 (9.4;11.6)	11.1 (9.8;12.8)	**0.003**
BMD total lumbar, g/cm2	1.2 (±0.2)	1.2 (±0.2)	0.868
ASMI, Kg/m2	8.2 (±1.3)	7.7 (±1.3)	**<0.001**
Sarcopenia (%)	4 (2.7%)	0 (0.0%)	**0.042**
HOMA-IR	2.0 (1.3;3.5)	2.0 (1.3;3.0)	0.681

Results are mean (±standard deviation), median (interquartile range) or N(%)

Supplemental Table 3. Unadjusted and adjusted means of ageing-related parameters according to smoking and HIV+ status

				Unadjusted mean (±SD)	Adjusted mean (±SE)
**FEV$_1$**	**Controls**	<10 PY		101.8 (±14.7)	101.7 (±1.7)
		>10 PY	Former smokers	103.3 (±17.9)	103.0 (±3.1)
			Current smokers	99.9 (±15.1)	99.9 (±2.5)
	**HIV**	<10 PY		100.2 (±14.8)	100.3 (±1.6)
		>10 PY	Former smokers	101.6 (±14.4)	101.2 (±3.1)
			Current smokers	93.6 (±21.6)	93.7 (±2.2)
**BMD Hip**	**Controls**	<10 PY		1.04 (±0.17)	1.04 (±0.02)
		>10 PY	Former smokers	1.04 (±0.08)	1.04 (±0.03)
			Current smokers	1.00 (±0.17)	1.02 (±0.02)
	**HIV**	<10 PY		1.01 (±0.16)	1.01 (±0.01)
		>10 PY	Former smokers	0.96 (±0.16)	0.96 (±0.03)
			Current smokers	0.93 (±0.13)	0.92 (±0.02)
**ASMI**	**Controls**	<10 PY		8.30 (±1.20)	8.24 (±0.11)
		>10 PY	Former smokers	8.68 (±1.24)	8.51 (±0.23)
			Current smokers	7.73 (±1.31)	8.15 (±0.17)
	**HIV**	<10 PY		7.96 (±1.40)	8.01 (±0.11)
		>10 PY	Former smokers	7.66 (±1.20)	7.52 (±0.20)
			Current smokers	7.34 (±1.16)	7.19 (±0.14)
**PWV**	**Controls**	<10 PY		10.5 (±1.6)	10.5 (±0.2)
		>10 PY	Former smokers	11.1 (±1.8)	10.9 (±0.3)
			Current smokers	10.9 (±1.7)	10.7 (±0.3)
	**HIV**	<10 PY		10.7 (±2.0)	10.7 (±0.2)
		>10 PY	Former smokers	11.4 (±3.2)	11.1 (±0.3)
			Current smokers	10.4 (±1.7)	10.6 (±0.2)
**HOMA-R**	**Controls**	<10 PY		2.95 (±4.13)	3.00 (±0.31)
		>10 PY	Former smokers	3.93 (±3.74)	3.82 (±0.55)
			Current smokers	2.34 (±1.71)	2.28 (±0.49)
	**HIV**	<10 PY		2.83 (±2.00)	2.85 (±0.30)
		>10 PY	Former smokers	3.75 (±4.28)	3.55 (±0.57)
			Current smokers	2.29 (±1.77)	2.38 (±0.40)
**eGFR** (Cockcroft)	**Controls**	<10 PY		101.5 (±25.2)	99.7 (±2.2)
		>10 PY	Former smokers	106.0 (±25.5)	107.1 (±4.0)
			Current smokers	103.0 (±24.8)	107.3 (±3.6)
	**HIV**	<10 PY		95.7 (±24.2)	96.2 (±2.2)
		>10 PY	Former smokers	96.2 (±30.0)	100.2 (±4.1)
			Current smokers	98.8 (±25.5)	95.7 (±3.0)

**Mixed effects linear regression model adjusted for age and gender*
SD: standard deviation; SE: standard error
